# ClassPass Memberships to Improve Well-Being Among Psychiatry Residents: A Pilot Randomized Controlled Trial

**DOI:** 10.1007/s40596-026-02384-y

**Published:** 2026-06-26

**Authors:** Kevin H. Yang, Letitia A. Mueller, Lauren D’Andrea, Kush V. Bhatt, Anaheed Shirazi, Desiree Shapiro, Neal Doran, Michael J. McCarthy, Sidney Zisook

**Affiliations:** 1 University of California San Diego School of Medicine, San Diego, CA USA; 2 VA San Diego Healthcare System, San Diego, CA USA

**Keywords:** Resident wellness, Physical activity, Anxiety, Depression, Burnout, Loneliness, Graduate medical education

## Abstract

**Objective:**

Resident physicians experience high rates of depression, anxiety, burnout, and loneliness, yet few evidence-based interventions have been evaluated in this population. This randomized controlled pilot trial examined the feasibility, acceptability, and preliminary effectiveness of ClassPass, a flexible fitness and wellness platform, to improve mental health outcomes among psychiatry residents.

**Methods:**

Twenty-eight psychiatry residents were randomized to receive a 16-week ClassPass membership (*n* = 14) or waitlist control (*n* = 14). Intervention participants received 20 ClassPass credits/month (equivalent to $45/month) to book activities of their choosing. Outcomes included measures of depression, anxiety, burnout, and loneliness, assessed at baseline and 16 weeks. Multivariable linear regression models evaluated intervention effects on change scores relative to the waitlist control, adjusting for baseline scores and demographics. Participant feedback was assessed post-intervention.

**Results:**

Twenty-eight of 56 eligible residents (50.0%) enrolled and were randomized; 26 (92.9%) completed the 16-week assessment. Participant engagement was high, with 76.9% reporting use of most or all credits, and 100% recommended program expansion. Qualitative themes highlighted appreciation for variety and flexibility, improved exercise habits, and enhanced social connection. The intervention was associated with a statistically significant reduction in anxiety (*p* = 0.012, *d* = 1.07), while improvements in depression (*p* = 0.059, *d* = 1.06) and loneliness (*p* = 0.062, *d* = 1.12) were not statistically significant. No significant difference was observed for burnout (*p* = 0.224).

**Conclusions:**

These findings suggest a flexible fitness platform is feasible, acceptable, and may reduce anxiety symptoms among psychiatry residents. High engagement and satisfaction suggest such platforms may overcome barriers common to traditional wellness interventions. Larger studies are needed to confirm these findings.

The demanding nature of residency training contributes to high rates of depression, anxiety, burnout, and loneliness among resident physicians [1–3]. Psychiatry residents may be particularly vulnerable given regular exposure to patients experiencing severe mental illness, trauma, and suicidality, with potential consequences for both well-being and professional satisfaction [4]. Despite recognition of these challenges, residents often struggle to prioritize self-care due to time constraints [5]. While physical activity, social interaction, and meditation have shown promise as potential interventions [6], traditional “one size fits all” wellness programs often fail to accommodate individual preferences, geographic constraints, and unpredictable schedules, leading to low participation and limited effectiveness [5, 7].

Flexible, technology-enabled wellness platforms, such as ClassPass [8], may offer a novel approach to these challenges by providing on-demand access to a network of fitness classes, gyms, and wellness services, allowing users to select activities based on individual preferences and potentially overcoming traditional barriers to wellness program participation. While some corporations have begun to incorporate such platforms as an employee benefit [9], no published randomized controlled trial (RCT) has examined their effectiveness among healthcare workers. We hypothesized that such a platform would improve mental health outcomes through multiple mechanisms: increased physical activity, which has established antidepressant and anxiolytic effects; social engagement through shared fitness activities with colleagues, which may reduce loneliness; and enhanced autonomy over self-care, which may buffer against burnout. This pilot RCT aimed to examine the feasibility, acceptability, and preliminary effectiveness of ClassPass for improving depression, anxiety, burnout, and loneliness among psychiatry residents.

## Methods

We conducted a pilot randomized controlled trial with a waitlist control design at the UC San Diego Department of Psychiatry between February 2025 and June 2025 to evaluate the feasibility, acceptability, and preliminary effectiveness of ClassPass as a wellness intervention for psychiatry residents. This study was approved by the UC San Diego Institutional Review Board and all participants provided informed consent.

Participants were recruited via department-wide email listservs and announcements during didactic sessions between February 1 and February 14, 2025. Inclusion criteria included current enrollment as a resident physician (training years 1–4), English proficiency, willingness to engage in wellness activities of their choosing, and access to a smartphone or computer. The sole exclusion criterion was an active ClassPass membership at enrollment. Of 56 eligible residents, 28 (50.0%) expressed interest and were enrolled and randomized.

Following the baseline assessment, participants were randomized 1:1 to either an immediate intervention group (*n *= 14) or a 16-week waitlist control group (*n* = 14). A 16-week intervention period was pre-specified in the study protocol to balance sufficient time for behavioral change and wellness habit formation, the spring academic year calendar, and available funding. Due to the nature of the intervention, participants could not be blinded to group assignment. However, all investigators involved in data analysis remained blinded throughout the study. Blinding was maintained by the study coordinator (LM), who was the sole individual with access to the randomization allocation. All survey data were de-identified prior to analysis.

Intervention participants received access to ClassPass [8], a commercial fitness platform providing on-demand access to a network of in-person and virtual wellness activities across more than 2500 cities globally, including San Diego. Activities include group fitness classes (e.g., yoga, Pilates, strength training, cycling), gym access, mindfulness-based activities, and restorative services (e.g., massage and other spa services), with scheduling managed through a mobile application. Participants were provided a fixed monthly credit allotment of 20 credits (valued at $45/month); at this tier, participants could book a variety of activities, including gym access (approximately 1 credit per visit), yoga or meditation (3–4 credits per session), and cycling or strength training (6–8 credits per session), allowing for approximately 3–6 sessions per month depending on activity type. Participants were free to self-select class type, frequency, and location, and no minimum attendance requirement was imposed, reflecting a pragmatic design intended to mirror real-world uptake of commercial fitness memberships.

Participants randomized to the control group maintained their usual wellness activities and did not receive access to the ClassPass platform during the 16-week study period. Upon study completion, control participants were offered a 12-week membership (also funded at $45/month) based on available funding, which was disclosed to all participants at the time of enrollment.

Four outcomes were pre-specified prior to study initiation based on their status as the most consistently documented mental health challenges among resident physicians: depression, anxiety, burnout, and loneliness. These outcomes were assessed via electronic surveys at baseline (Week 0) and post-intervention (Week 16). Depression was assessed using the Patient Health Questionnaire-9 (PHQ-9), a 9-item measure of depressive symptom severity over the past 2 weeks (range 0–27), with excellent internal consistency, test-retest reliability, and sensitivity to change [10]. Anxiety was assessed using the Generalized Anxiety Disorder-7 (GAD-7), a 7-item measure of anxiety severity over the past 2 weeks (range 0–21), with excellent psychometric properties [11]. Burnout was assessed using the Single-Item Burnout Scale, a single-item measure (range 1–5) validated against the Maslach Burnout Inventory emotional exhaustion subscale in physician populations [12]. Loneliness was assessed using the UCLA Loneliness Scale-3 (UCLA-3), a 3-item measure (range 3–9), with good internal consistency and construct validity [13]. At baseline, participants also reported demographic characteristics (age, gender, race/ethnicity, training year) and current use of gym or fitness memberships.

At the 16-week assessment, intervention participants completed additional items assessing program experience, including one item on credit usage and ten items on program perceptions using a 5-point Likert scale across domains including well-being impact, convenience, variety of activities, social connection with colleagues, and recommendation for program expansion. Two open-ended questions solicited feedback regarding overall experience and areas for improvement.

Baseline characteristics were summarized using means and standard deviations for continuous variables and frequencies and percentages for categorical variables. Program feedback Likert-scale items were summarized descriptively, and open-ended responses were reviewed using thematic analysis, in which responses were read iteratively and coded to identify recurring themes. Multivariable linear regression models were used to evaluate intervention effects on 16-week change scores (such that positive values indicated symptom improvement) between the intervention and waitlist control groups. Separate models were fit for each outcome, adjusted for the respective baseline outcome score, age, sex, race/ethnicity, and training year. Mean differences between groups with standard errors and Cohen’s *d* effect sizes were derived from each model. Analyses utilized a complete-case approach. Statistical significance was set at *p* < 0.05. Given the exploratory nature of this pilot study, no adjustments were made for multiple comparisons. Analyses were conducted using Stata 18.0 (StataCorp, College Station, TX).

## Results

Of 56 psychiatry residents in the program, 28 (50.0%) expressed interest and were enrolled and randomized to the active intervention (*n* = 14) or waitlist control (*n* = 14) groups. Two participants (one per group) did not complete the 16-week assessment and were considered lost to follow-up, resulting in 26 participants (92.9%) completing the study. Among all enrolled participants, the mean age was 31.1 years, the majority were female (67.9%), and participants were predominantly White Non-Hispanic (60.7%). Training levels were evenly distributed across training years 1–3 (28.6% each), with fewer fourth-year residents (14.3%). Mean baseline scores were as follows: PHQ-9 = 4.4 (SD = 3.0), GAD-7 = 5.3 (SD = 3.4), Single-Item Burnout Scale = 2.8 (SD = 0.7), and UCLA-3 = 5.1 (SD = 1.3). Participant characteristics by group are presented in Table 1.

**Table 1:** Baseline characteristics of psychiatry residents enrolled in the ClassPass study

Characteristic	ClassPass intervention (*n* = 14)	Waitlist control (*n* = 14)
Age, mean (SD)	32.5 (3.0)	29.8 (2.3)
Gender, ***n*** (%)		
Male	7 (50.0)	2 (14.3)
Female	7 (50.0)	12 (85.7)
Race/ethnicityᵃ, ***n*** (%)		
White	9 (64.3)	8 (57.1)
Asian	5 (35.7)	6 (42.9)
Hispanic	0 (0.0)	2 (14.3)
Otherᵇ	0 (0.0)	1 (7.1)
Training year, ***n*** (%)		
Year 1	4 (28.6)	4 (28.6)
Year 2	3 (21.4)	5 (35.7)
Year 3	6 (42.9)	2 (14.3)
Year 4	1 (7.1)	3 (21.4)
Baseline wellness practices, ***n*** (%)		
Active gym/fitness memberships	9 (64.3)	10 (71.4)
Baseline outcome measures, mean (SD)		
Depression (PHQ-9; 0–27)	4.7 (2.4)	4.0 (3.4)
Anxiety (GAD-7; 0–21)	5.1 (3.0)	5.5 (3.7)
Burnout (Single-Item Burnout Scale; 1–5)	2.8 (0.8)	2.7 (0.7)
Loneliness (UCLA Loneliness Scale-3; 3–9)	4.6 (1.2)	5.6 (1.3)

Abbreviations: *GAD-7*, Generalized Anxiety Disorder-7; *PHQ-9*, Patient Health Questionnaire-9; *SD*, standard deviation

ᵃRace/ethnicity categories were not mutually exclusive; percentages may sum to > 100%

ᵇOther includes Black/African American, American Indian/Alaska Native, Middle Eastern/North African, and Native Hawaiian/Pacific Islander, which were collapsed due to small cell sizes

Regarding program feedback, intervention group participants (*n* = 13) reported high satisfaction, with 76.9% reporting use of most or all of their available activity credits. Notably, 100% of participants agreed or strongly agreed that the service should be available to all residents at UC San Diego and the majority endorsed positive impacts on well-being, stress management, and convenience. Qualitative analysis of open-ended responses (*n* = 7) further supported these findings, with participants highlighting the variety and flexibility of offerings, improvements in exercise habits, and enhanced social connection with co-residents through shared fitness activities (Table 2). The most common suggestion for improvement was increasing the monthly credit allotment.

**Table 2:** Qualitative themes from open-ended feedback

Theme	*n* (%)^a^	Description	Representative quotes
Variety and flexibility	3 (42.9%)	Participants appreciated the range of activities and ease of exploring new options	“The fact that it was free gave me the courage to try out these group classes…something I felt intimidated by before.” “ClassPass has so many options and it’s so easy and convenient to use.”
Improved exercise habits	4 (57.1%)	Participants reported establishing new routines and increased physical activity	“I went from exercising zero times a week to a few times a week, and I now have a routine.” “It has helped with my ability to maintain physical activity.”
Enhanced social connection	2 (28.6%)	Shared participation in fitness activities with co-residents (e.g., attending classes together) facilitated social bonding	“I was able to see co-residents that I normally do not see and do something productive through exercising together.” “[Making] fitness plans with co-residents…improved my feelings of closeness with them.”
High perceived program value	7 (100%)	Participants strongly endorsed the program's positive impact on well-being and recommended its expansion to all residents	“Out of all the wellness activities so far, this has made by far the biggest positive impact on my life. I strongly believe that every resident should have access to ClassPass, and that the residency should allocate money here.” “Super valuable program to improve wellness….Would recommend it's available to all residents.”
Suggestions for improvement	4 (57.1%)	Participants suggested increasing credit allotments and adding coordination support to optimize the program	“Not enough ClassPass credits…We need more money to be able to consistently go to class every week.” “It would be helpful to have a point person to coordinate class meet ups.”

^a^*n* (%) reflects the number of participants who provided open-ended responses (*n* = 7)

Regarding intervention effects, the active intervention was associated with a statistically significant reduction in anxiety symptoms (GAD-7 mean difference = 2.75, SE = 0.99, *p* = 0.012, *d* = 1.07). Improvements in depression (PHQ-9 mean difference = 2.65, SE = 1.32, *p* = 0.059, *d* = 1.06) and loneliness (UCLA-3 mean difference = 1.59, SE = 0.80, *p* = 0.062, *d* = 1.12) were not statistically significant. No significant difference was observed for burnout (Single-Item Burnout Scale mean difference = 0.35, SE = 0.28, *p* = 0.224, *d* = 0.47).

## Discussion

This pilot RCT with a waitlist control group demonstrates that a flexible, technology-enabled wellness platform is feasible and highly acceptable among psychiatry residents, with preliminary data suggesting a potential benefit for anxiety symptoms. To our knowledge, this is the first RCT examining a commercial fitness membership platform among resident physicians. Enrollment represented 50% of the eligible resident pool, supporting feasibility, and retention was 92.9%, with 100% of intervention participants recommending program expansion, supporting acceptability. These findings suggest that flexible wellness interventions allowing users to choose activities based on individual preferences, geographic availability, and scheduling needs may be well-suited to the unique demands of residency training.

Participant engagement and satisfaction were high, with all intervention participants recommending program expansion and the majority endorsing positive impacts on their well-being. Qualitative responses highlighted three key themes: appreciation for the variety and flexibility of class offerings, improvements in exercise habits, and enhanced social connection with co-residents through shared fitness activities. The platform’s flexibility may have reduced barriers to wellness engagement by allowing participants to select activities fitting their often-changing schedules and availability. Indeed, the majority of participants (76.9%) reported utilizing most or all of their available credits, suggesting sustained engagement throughout the 16-week intervention period. At $45 per resident per month (approximately $2520 total for the 14-person intervention group over 16 weeks), this intervention represents a modest investment compared to other evidence-based wellness programs such as mindfulness-based stress reduction courses, which can cost several hundred dollars per participant [14], and may thus be scalable as a departmental benefit. The ClassPass Corporate program offers centralized enrollment and employer engagement tracking, and has been adopted by major corporations including Google and Morgan Stanley [9], suggesting feasibility at an organizational level. Finally, participants suggested increasing the monthly credit allotment; as 20 credits represented the lower end of available membership tiers, this may have limited participation frequency for some residents and should be examined in future studies.

The significant reduction in anxiety symptoms observed in the intervention group is consistent with meta-analytic evidence demonstrating medium effects of physical activity across diverse adult populations, including protective effects against anxiety development over time [15]. Beyond the direct physiological benefits of exercise, the ClassPass platform may have also reduced anxiety by providing participants with a sense of autonomy and control over their self-care. These findings thus suggest that flexible, self-directed fitness platforms may offer a particularly well-suited approach to anxiety reduction among psychiatry residents, for whom autonomy and schedule flexibility are key barriers to wellness engagement.

Although improvements in depression and loneliness were not statistically significant, the large effect sizes (*d* = 1.06 and *d* = 1.12, respectively) [16] suggest potential clinical relevance that may be detectable with a larger sample. These trends are consistent with evidence that physical activity and social engagement can improve depressive symptoms and mitigate loneliness [17, 18]. As noted in qualitative responses, the social component of the platform may have contributed to reductions in loneliness through shared participation in fitness activities with co-residents, rather than a general sense of programmatic support. Interestingly, the intervention did not significantly reduce burnout, which may reflect an important conceptual distinction: anxiety, depression, and loneliness are state-level symptoms that may be more readily modifiable through individual behavioral interventions, whereas burnout is increasingly conceptualized as a systemic occupational phenomenon driven by workload, limited autonomy, and inadequate organizational support that individual-level wellness programs are unlikely to remediate in isolation [19, 20]. Notably, baseline symptom severity was in the low-to-mild range, potentially creating floor effects that limited the ability to detect significant changes. Future studies including residents with higher baseline symptoms may yield more interpretable outcomes.

Our study should be interpreted in the context of several limitations. First, the small sample size limits statistical power, and four outcomes were assessed without adjustment for multiple comparisons, increasing the risk of Type 1 error; findings should thus be interpreted with caution. Second, this study was conducted at a single institution among psychiatry residents in a large metropolitan area, limiting generalizability to other specialties, geographic regions, and settings with fewer available studios. Third, the study relied on self-reported measures, which may be subject to response and recall bias. Fourth, despite their established validity, the single-item burnout measure and UCLA-3 may have reduced sensitivity to detect changes; future studies might benefit from more comprehensive measures such as the full Maslach Burnout Inventory and UCLA 20-item Loneliness Scale. Fifth, objective platform utilization data were unavailable, and we did not assess which specific activities participants selected, though this was intentional, as a key feature of the intervention was allowing participants to select activities based on their individual preferences. Sixth, self-selection bias may limit generalizability, as enrolled residents may have had pre-existing interest in wellness, potentially limiting applicability to less wellness-engaged residents who may be most in need of support. Seventh, the waitlist control design did not account for placebo or expectancy effects, and the 12-week post-study control membership asymmetry, while not affecting the primary comparison, is acknowledged as a design limitation. Finally, the 16-week follow-up limits assessment of long-term benefit.

In summary, this pilot RCT provides preliminary evidence that a flexible, technology-enabled wellness platform is feasible, highly acceptable, and may reduce anxiety symptoms among psychiatry residents. The effect sizes observed will inform power calculations for future adequately powered trials, which should include longer follow-up periods, more nuanced burnout measures, active control conditions, and higher credit allotments. Such studies may help inform scalable, effective wellness programs across psychiatry and other specialties to support resident well-being.

## Data Availability

The datasets generated and analyzed during the current study are available from the corresponding author on reasonable request.
